# Integrated evaluation of Nigrosome 1 sign, neuromelanin-sensitive MR and iron deposition

**DOI:** 10.1007/s11604-025-01858-7

**Published:** 2025-08-18

**Authors:** Yasutaka Fushimi, Kiarash Ghassaban, Sean K. Sethi, Satoshi Nakajima, Sachi Okuchi, Akihiko Sakata, Takayuki Yamamoto, Sayo Otani, Satoshi Ikeda, Yuta Terada, Atsushi Shima, Nobukatsu Sawamoto, Yuji Nakamoto

**Affiliations:** 1 Department of Diagnostic Imaging and Nuclear Medicine, Graduate School of Medicine, Kyoto University, Kyoto, Japan; 2 SpinTech MRI., Bingham Farms, MI USA; 3 Department of Neurology, Kyoto University Hospital, Kyoto, Japan; 4 Department of Human Health Sciences, Graduate School of Medicine, Kyoto University, Kyoto, Japan

**Keywords:** Nigrosome 1, Neuromelanin sensitive MRI, Quantitative susceptibility mapping, Parkinson’s disease

## Abstract

**Purpose:**

To differentiate between Parkinson’s Disease (PD) and healthy controls by using integrated analysis of PD-specific MR findings including deformation of the substantia nigra pars compacta (SNpc), signal loss in neuromelanin (NM) sensitive MRI, and iron deposition in the deep gray matter (DGM) structures.

**Materials and methods:**

Patients with PD and healthy controls were recruited between August 2022 and December 2023. All subjects underwent 3 T MRI including a magnetization transfer contrast (MTC) and a double flip angle multi-echo protocol as part of Strategically Acquired Gradient Echo (STAGE). The data analysis included detecting the presence of Nigrosome-1 (N1) sign in the SNpc, signal intensity and volume of NM content and iron quantification through quantitative susceptibility mapping (QSM) in DGMs. The 3D regions of interest were manually demarcated on QSM maps. Mean susceptibility values from global analysis (whole structure) as well as regional high iron analysis (age-based threshold) were extracted for each individual structure. Univariate and multivariate analyses were performed using these parameters.

**Results:**

Nineteen patients with PD (68.0 ± 8.0 years, 10 males, Hoehn and Yahr scale 1 (n = 1), 2 (n = 13), 3 (n = 4), 4 (n = 1)) and 21 healthy controls (68.3 ± 8.6 years, 12 males) were enrolled. Discriminating PD from controls was successful using each method: N1 sign (P < 0.001), NM volume (P < 0.001), susceptibility values of global analysis (caudate, P < 0.001; putamen, P < 0.001; pulvinar, P = 0.006), regional analysis (putamen, P < 0.001; pulvinar, P = 0.009, thalamus, P = 0.008). Stepwise logistic regression analyses were performed, and the best model was created using N1 sign, NM volume, regional analysis (putamen, red nucleus and thalamus) (area under the curve of 0.99).

**Conclusions:**

Integrated analysis of PD specific MR findings including N1 sign, NM volume, and iron content in the DGM structures robustly discriminates between PD and healthy controls.

**Supplementary Information:**

The online version contains supplementary material available at 10.1007/s11604-025-01858-7.

## Introduction

Progressive loss of dopaminergic neurons, which contain pigments called neuromelanin in the substantia nigra (SN) is a characteristic of Parkinson’s disease (PD) [1, 2]. Symptoms of PD are thought to appear after 50–60% of dopamine neurons have degenerated, and the presymptomatic phase often spans more than 20 years [3, 4]. Clusters of dopamine-containing neurons in calbindin-poor zones in the substantia nigra are termed Nigrosomes. Nigrosome-1 (N1), located in the caudal and posterolateral part of the substantia nigra pars compacta, is the largest dopamine-containing cluster and is mostly affected by PD [5]. The N1 has been described as hyperintense on iron-sensitive SWI sequences, resembling a swallow tail [6], and the presence of N1 sign or a swallow tail sign has been shown to be sensitive and specific with a high negative predictive value in PD at 3 T MR. However, some controversy exists regarding the interpretation of N1 signal intensity [7]: Studies comparing T2*WI with histology have shown that T2*WI hyperintense region corresponds to N1 on histology [8]. However, Brammerloh et al. [7] demonstrated that N1 as seen with histological techniques was significantly thinner and longer compared to the hyperintense region on T2*WI, with a distinct shape (i.e. flat and disk-like) in histology versus ovoid-shaped on T2*WI. They concluded that N1 appears as a low signal intensity region on T2*WI. Furthermore, Brammerloh et al. [9] reported that N1 exhibits high R2* values, which are attributed to the iron bound to neuromelanin inside dopaminergic neurons. Characterization of the appearance of N1 and a set of rules for the presence or absence or partial presence of any N1 sign are of crucial importance. Therefore, sufficient in-plane resolution, slice thickness and signal-to-noise (SNR) are required to image the SN efficiently [10].

In addition to the N1 sign, neuromelanin-sensitive MRI (NM-MRI) methods have been investigated for the early diagnosis of PD [11–15]. Neuromelanin-iron complexes have T1-shortening effects, and magnetization transfer (MT) effects also contribute to the MR contrast by suppressing signals from background brain parenchyma [11, 16–19]. Various types of NM-MRI have been investigated to gain better intensity of neuromelanin, including various MT pulses with flip angles [16], denoising approaches [15] as well as reproducibility for performing NM-MRI experiments with high quality [20]. Although some reports suggest that NM-MRI may be sensitive to proton density, while not likely to be sensitive to NM and metal content [21, 22], NM-MRI still offers a great potential to reflect human dopaminergic and noradrenergic function in vivo, making it a clinically promising biomarker [21, 23, 24].

Deformation of the substantia nigra pars compacta (SNpc) (i.e. loss of swallow tail sign), signal loss on NM-MRI, and iron deposition in the deep gray matter (DGM) structures are important findings in PD. Since non-heme iron content in DGM structures corelate with age [25, 26], susceptibility values can be evaluated with consideration of whole-structural (global) iron as a function of age. Previous studies established the importance of threshold at a given age and for each DGM structure, respectively [26–28]. Susceptibility values of these thresholded areas can be used for regional analysis where only the average of susceptibility values higher than an age- and structure-dependent threshold contributes to the susceptibility-age relationship [26–28]. Using thresholded or above-normal range iron not only is a more robust predictor of iron with age, but can help discriminate between PD and healthy control (HC) better than using whole-region approaches which have variance in spatial distribution of iron.

N1 sign, NM volume, and susceptibility values of DGM structures have usually been measured separately in the evaluation of PD [29], however, a limited number of integrated analysis including these measures has been reported [30]. By selecting appropriate measurement methods, useful imaging biomarkers may be found for the evaluation of PD. The aim of this study was to effectively differentiate between PD and HCs by using integrated analysis of PD-specific MR findings as N1 sign, NM volume, and iron content in the DGM.

## Materials and methods

### Subjects

This study was approved by the local institutional review committee. Patients with PD and healthy controls were recruited between August 2022 and December 2023, and all subjects gave written consent for this prospective study. Inclusion criteria for patients with PD were a clinically established or probable diagnosis of PD and consent to undergo additional MR scans as part of this study. The inclusion criteria for HC were the absence of any current diseases, including neurological disorders.

For patients with PD, the on-state and off-state scores of the MDS-UPDRS Part III (MDS-UPDRS3) were collected from all participants [31].

### MR imaging

All subjects underwent 3 T MRI (MAGNETOM Skyra, Siemens Healthineers, Erlangen, Germany) including a magnetization transfer contrast (MTC) and a double flip angle multi-echo protocol as part of Strategically Acquired Gradient Echo (STAGE) (SpinTech-MRI., Bingham Farms, MI, USA). These images were acquired along the along the anterior commissure–posterior commissure (AC–PC) line. The imaging parameters are shown in Tables 1 and 2 [32].

**Table 1:** The Imaging parameters of the two MTC sequences separated by their flip angles

	MTC 12	MTC 30
Orientation	Axial	Axial
TR (ms)	62	62
TE (ms)	7.5/15/22.5/30/37.5	7.5/15/22.5/30/37.5
MTC	On	On
FA (degree)	12	30
Base resolution	384	384
Slice thickness (mm)	1.34	1.34
FOVread × FOVphase (mm^2^)	256 × 192	256 × 192
Slice gap (%)	20	20
Slice thickness (mm)	1.34	1.34
Number of slices	48	48
Voxel size (mm^3^)	0.7 × 0.7 × 1.34	0.7 × 0.7 × 1.34
Interpolation	Off	Off
Acceleration factor	GRAPPA 2 ×	GRAPPA 2 ×
Acquisition time	4 m 31 s	4 m 31 s

*MTC* magnetization transfer contrast

**Table 2:** The Imaging parameters of STAGE SWI sequences separated by flip angles

	HR SWI PDW	HR SWI T1W
Orientation	Axial	Axial
TR (ms)	29	29
TE (ms)	7.5/15/22.5	7.5/15/22.5
TI (ms)	N/A	N/A
FA (degree)	6	27
Base resolution	384	384
FOVread × FOVphase (mm^2^)	256 × 192	256 × 192
Slice gap (%)	0	0
Slice thickness (mm)	1.34	1.34
Number of slices	112	112
Voxel size (mm^3^)	0.67 × 1.00 × 1.34	0.67 × 1.00 × 1.34
Interpolation	Off	Off
Acceleration factor	GRAPPA 2 ×	GRAPPA 2 ×
Acquisition time	4 m 48 s	4 m 48 s

The imaging parameters of double flip angle multi-echo protocol as part of Strategically Acquired Gradient Echo (STAGE) are shown. The low and high flip angle sequences have been marked with proton density weighted (PDW) and T1-weighted (T1W), respectively

*GRAPPA* GeneRalized autocalibrating partially parallel acquisitions

### Postprocessing procedure

Detection of the presence of Nigrosome-1 (the N1 sign) in the SNpc was performed as previously described by referring to specific patterns of normal-appearing -N1 variants by Cheng et al. [10] (Supplementary Fig. 1). We assessed the appearance of N1 sign with true-SWI (tSWI), which is reconstructed using the same post-processing pipeline as the SWI but with QSM as the mask instead of filtered phase information. This technique avoids geometry-induced phase variation by using the source magnetic susceptibility [33]. The N1 sign was initially assessed by one rater (K.G.) with over 10 years of relevant experience, and subsequently confirmed by a board-certified radiologist (Y.F.).

**Fig. 1:**
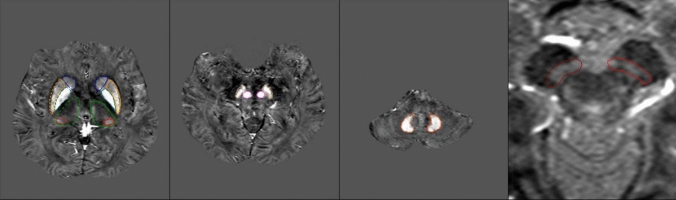
Representative ROIs are shown. DGM including *CN* caudate nucleus, *GP* globus pallidus, *PT* putamen, *TH* thalamus, *Pul* pulvinar thalamus, *SN* substantia nigra, *RN* red nucleus, *DN* dentate nucleus, *NM* neuromelanin

Signal intensity and volume of NM content were drawn and analyzed on MTC images while iron quantification was performed through STAGE-QSM in DGM including caudate nucleus (CN), globus pallidus (GP), putamen (PT), thalamus (TH), pulvinar thalamus (Pul), substantia nigra (SN), red nucleus (RN), and dentate nucleus (DN). The 3D regions of interest (ROI) were manually demarcated on MTC and QSM maps by the postprocessing team at SpinTech MRI, Inc. and confirmed by an author (K.G.) with over 10 years of relevant experience and following the same technique described in detail by He et al. [30]. Representative ROIs are shown in Fig. 1. Since non-heme iron content in DGMs correlates with age, magnetic susceptibility values were evaluated with whole-structure (global) and regional (thresholded) approaches. The upper 95% prediction interval derived from the previous results concerning the relationship between global mean susceptibility vs. age were used as the threshold at a given age and for each DGM structure [26–28]. Thus, we also obtained mean susceptibility values from global analysis (i.e. from the whole structure) as well as regional high iron analysis (extracted from thresholding susceptibility values higher than the upper 95% prediction intervals at a given age).

We have evaluated differences of susceptibility values associated with the laterality of symptoms (Supplementary Annex).

### Statistical analysis

To differentiate between PD and HC, univariate analyses were performed for each of above-mentioned parameters: (i) N1 sign, (ii) NM signal intensity and NM volume, (iii) mean susceptibility values of DGMs (caudate nucleus (CN), globus pallidus (GP), putamen (PT), thalamus (TH), pulvinar thalamus (Pul), substantia nigra (SN), red nucleus (RN), and dentate nucleus (DN)) as well as regional high susceptibility values of DGMs. Two sample t-test was performed for these parameters (i), (ii), and (iii), respectively, to differentiate between PD and HC. When datasets did not follow a normal distribution, Mann–Whitney test was employed. Stepwise regression analysis was also performed using these parameters. Finally, the best model was chosen from (i), (ii), (iii) and the integrated model generated from stepwise regression analysis followed by an receiver operating characteristic (ROC) curve analysis. DeLong test was also performed to compare the performance of each model in differentiating between PD and HC.

Comparison of NM volume between the presence and absence of N1 sign was performed using Welch's test.

Additionally, these values were compared to the on-state and off-state MDS-UPDRS3 scores. Comparisons were performed using Pearson’s test, and Spearman’s rho was applied when the dataset did not follow a normal distribution.

These analyses were performed with Medcalc software version 20 (MedCalc Software, Ostend, Belgium) and P < 0.05 was considered as statistically significant. The plots and corresponding approximate linear regression lines were generated using R [34] and RStudio (Posit Software, PBC, Boston, MA, United States).

## Results

### Subjects

Nineteen patients with PD (68.0 ± 8.0 years, 10 males, Hoehn and Yahr scale 1 (n = 1), 2 (n = 13), 3 (n = 4), 4 (n = 1)) and 21 healthy controls (68.3 ± 8.6 years, 12 males) were enrolled. No participants were excluded from this study.

For patients with PD, MDS-UPDRS3 scores were obtained in both the on-state and off-state for all participants, except for one patient whose off-state score was unavailable (on-state score: 60). The mean ± standard deviation of the scores was as follows: on-state, 28.3 ± 15.9; off-state, 40.9 ± 12.0. The disease duration of PD was 12.0 ± 7.0 years.

### ROI analysis

**Fig. 2:**
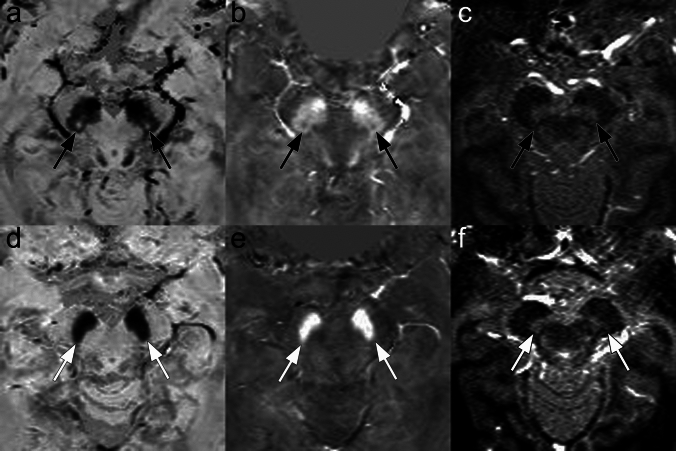
A 71-year-old male, HC (upper row, a, b, c). Representative images of tSWI (**a**) and QSM (**b**) showing the N1 sign as hyperintense and hypointense signal intensity, respectively, as well as MTC (**c**) showing the NM content in the midbrain area (black arrows). Note that QSM and tSWI were used for evaluation of N1, resulting in positive N1 sign in this case (type F from Supplementary Fig. 1). A 67-year-old female with PD (bottom row, **d**, **e**, **f**). Representative images of tSWI (**d**), and QSM (**e**) showing and absence of the N1 sign (white arrows). MTC (**f**) shows the degeneration of the NM content in the midbrain area (white arrows)

Representative images for N1 sign and NM-MRI are shown (Fig. 2). Overall results are shown in Table 3.

**Table 3:** Overall results of the ROI analyses

	PD (N = 19)	Control (N = 21)	P value
*SWI*			
N1 sign [yes/no]	[3/16]	[18/3]	P < 0.001*
*MTC*			
Neuromelanin volume [mm^3^]	251.3 [216.5, 279.2]	322.2 [286.8, 371.1]	P < 0.001*
*Global analysis (QSM)*			
CN [ppb]	28.9 [24.9, 38.0]	37.7 [32.1, 44.2]	P < 0.001*
GP [ppb]	114.7 [98.5, 130.7]	105.2 [100.7, 125.8]	P = 0.35
PT [ppb]	35.2 [25.1, 49.1]	57.9 [40.1, 69.8]	P < 0.001*
TH [ppb]	− 12.0 [− 14.7, − 7.8]	− 11.5 [− 14.8, − 8.5]	P = 0.95
Pul [ppb]	16.1 [7.9, 25.3]	29.3 [11.5, 44.5]	P = 0.006*
RN [ppb]	102.4 [73.3, 126.6]	109.8 [92.2, 130.5]	P = 0.32
SN [ppb]	125.7 [110.8, 140.7]	125.3 [113.1, 141.0]	P = 0.74
DN [ppb]	88.5 [66.5, 105.2]	81.2 [67.7, 97.3]	P = 0.51
*Regional analysis (QSM)*			
CN [ppb]	94.8 [87.1, 97.5]	96.8 [89.0, 101.6]	P = 0.14
GP [ppb]	215.1 [196.5, 260.9]	214.3 [195.5, 246.2]	P = 0.52
PT [ppb]	117.1 [108.4, 122.6]	133.3 [121.2, 145.2]	P < 0.001*
TH [ppb]	21.7 [17.1, 25.7]	26.2 [20.3, 32.5]	P = 0.008*
Pul [ppb]	71.2 [70.0, 78.4]	77.8 [72.0, 84.1]	P = 0.009*
RN [ppb]	191.0 [181.4, 204.0]	191.0 [181.6, 197.2]	P = 0.90
SN [ppb]	229.5 [211.2, 247.7]	222.4 [212.2, 231.8]	P = 0.16
DN [ppb]	176.9 [164.1, 190.0]	175.2 [170.0, 183.6]	P = 0.88

Note that 25- and 75-percentiles are shown in the brackets

*N1* Nigrosome 1, *CN* caudate nucleus, *GP* globus pallidus, *PT* putamen, *TH* thalamus, *Pul* pulvinar thalamus, *SN* substantia nigra, *RN* red nucleus, *DN* dentate nucleus

^*^Represents statistical significance (P < 0.05)

ROI analysis revealed significant differences between patients with PD and HC: N1 sign (P < 0.001), NM volume (P < 0.001), susceptibility values of global analysis (CN, P < 0.001; PT, P < 0.001; Pul, P = 0.006), regional analysis (PT, P < 0.001; Pul, P = 0.009, TH, P = 0.008) (Table 3).

### Univariate analysis

Discriminating PD from controls was successful using each method: N1 sign (P < 0.001), NM volume (P < 0.001), susceptibility values of global analysis (CN, P < 0.001; PT, P < 0.001; Pul, P = 0.006), regional analysis (PT, P < 0.001; Pul, P = 0.009, TH, P = 0.008).

### Multivariate analysis

**Fig. 3:**
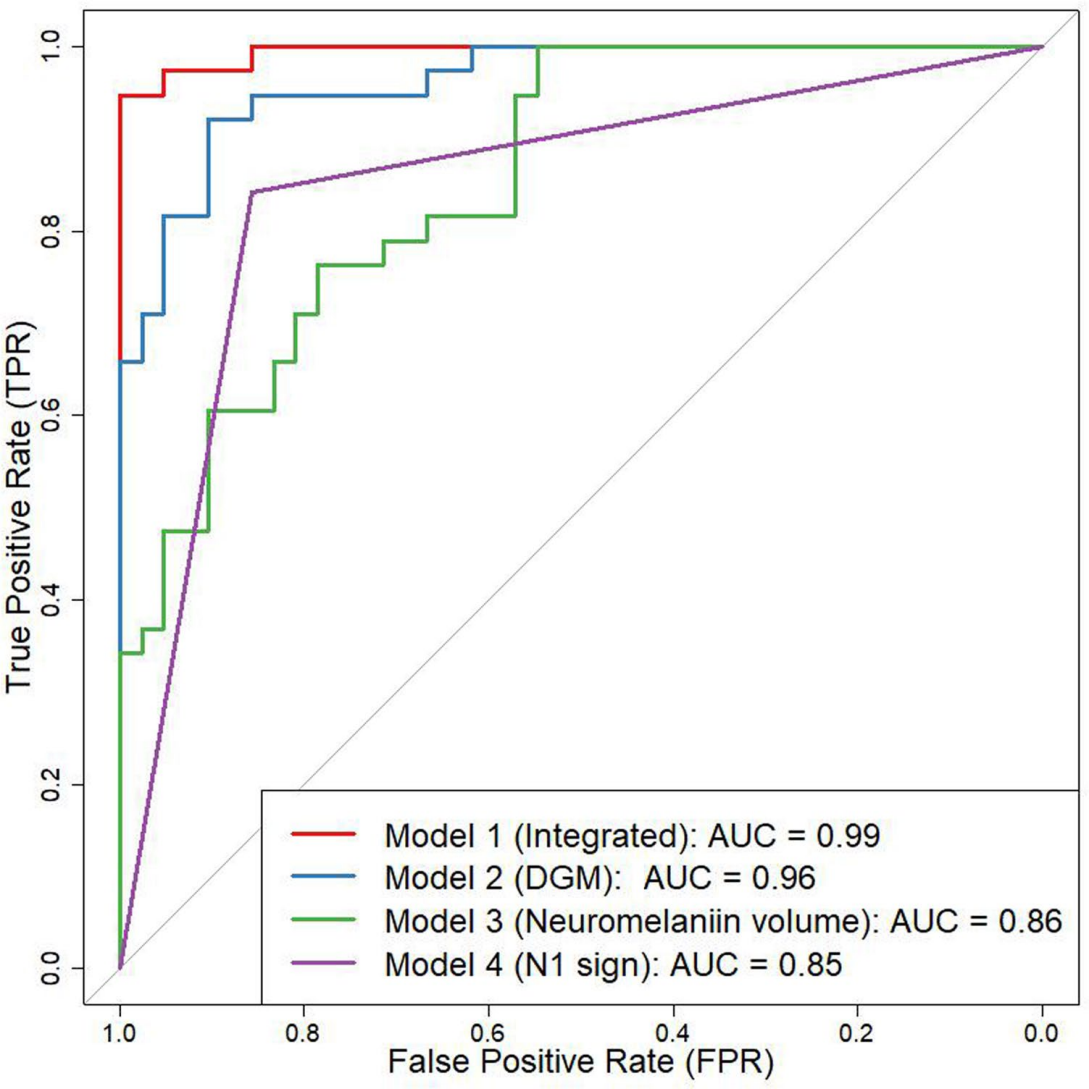
The ROC curve of the best model for differentiation between PD and healthy controls is shown. The best model was determined after stepwise logistic regression analyses. The best model used N1 sign, neuromelanin volume, susceptibility values of PT, RN and TH (AUC of 0.99). *ROC* Receiver Operating Characteristic, *N1* Nigrosome-1, *PT* putamen, *RN* red nucleus, *TH* thalamus

Models for distinguishing patients with PD from HC were created for (i) N1 sign, (ii) NM volume, and (iii) regional analysis of DGM, with areas under the curve (AUCs) values of 0.85, 0.86, and 0.96, respectively. Stepwise logistic regression analyses were conducted, and the optimal integrated model for distinguishing patients with PD from HC was developed using the N1 sign, NM volume, and regional analysis of PT, RN, and TH, achieving AUC of 0.99 (Fig. 3). The DeLong test revealed statistical significance with Bonferroni correction between the integrated model and (i) (P = 0.0013), as well as between the integrated model and (ii) (P = 0.0021).

### N1 sign and neuromelanin volume

Box and whisker plots of NM volume are presented for individuals categorized by the presence or absence of the N1 sign (yes/no) and for patients with PD as well as HC (Supplementary Fig. 2). Subjects with a positive N1 sign (n = 35, 328.5 ± 57.9 mm^3^), exhibited larger NM volumes compared with those with a negative N1 sign (n = 37, 250.7 ± 40.6 mm^3^) (P < 0.001).

### MDS-UPDRS3 scores

**Fig. 4:**
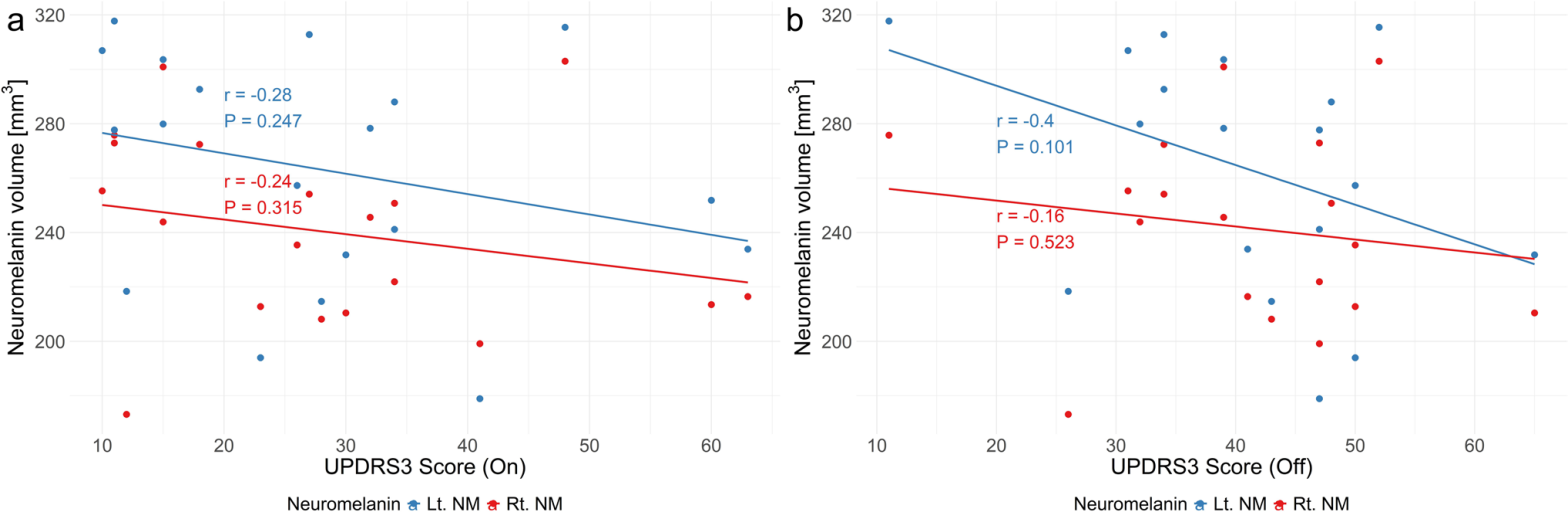
Neuromelanin (NM) volumes compared against with MDS-UPDRS3 scores of on-state (**a**) and off-state (**b**). Neuromelanin volumes were not correlated with either of MDS-UPDRS3 scores. Note that the off-state score for one patient, whose on-state score was 60, was not recorded

N1 sign was positive for 3 out of 19 patients, whose MDS-UPDRS3 scores were as follows (on-state/off-state): 11/11; 34/48; and 48/52, respectively. NM volumes were not correlated with either the on-state or off-state MDS-UPDRS3 scores (Fig. 4).

The relationship between susceptibility values from global or regional analysis and the on-state or off-state MDS-UPDRS3 scores is shown in Supplementary Fig. 3.

**Fig. 5:**
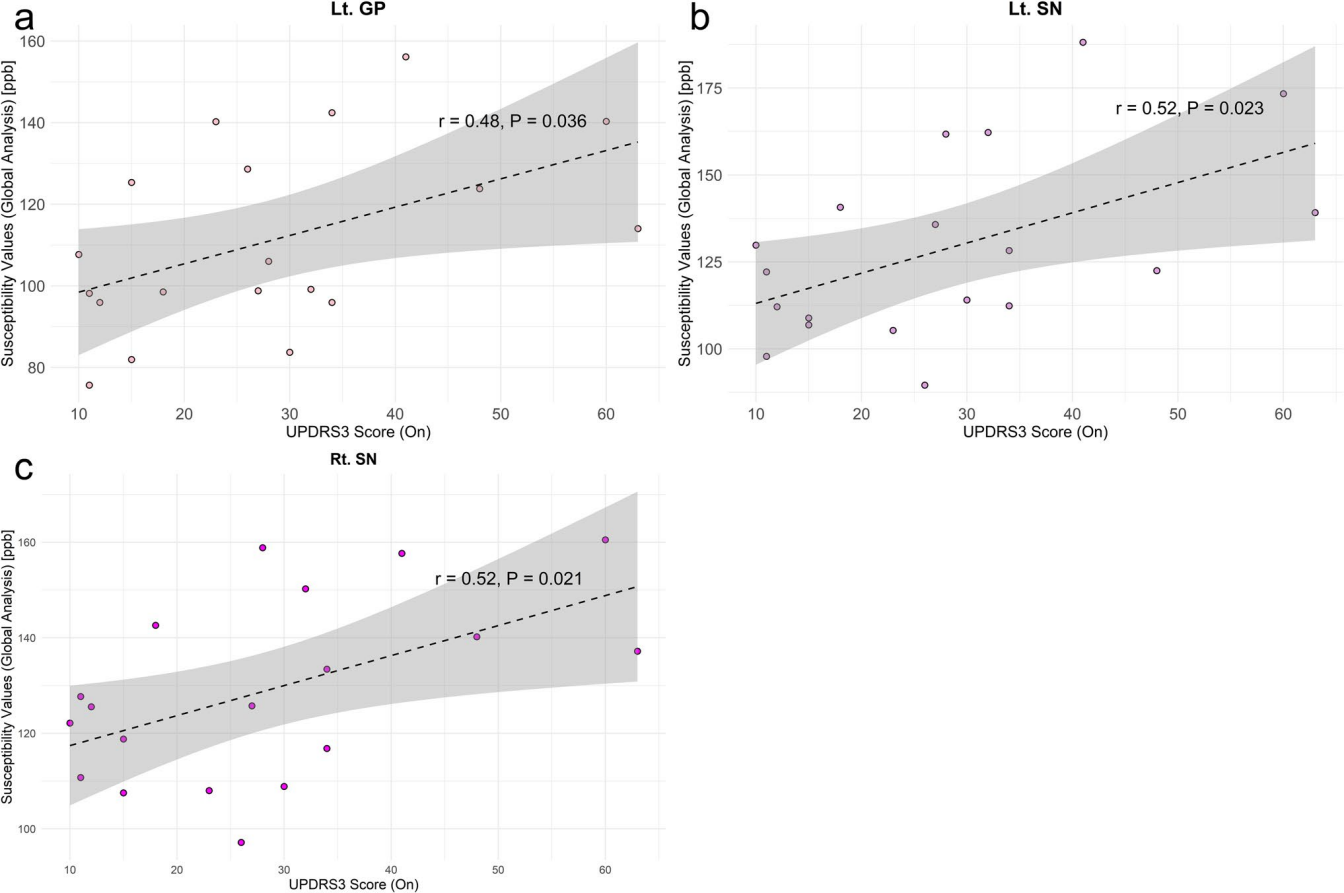
The relationship between global susceptibility values and MDS-UPDRS3 scores showing the on-state MDS-UPDRS3 score significantly correlated with **a** Lt. GP (r = 0.48, P = 0.036), **b** Lt. SN (r = 0.52, P = 0.023), and **c** Rt. SN (r = 0.52, P = 0.021). No susceptibility values were correlated with the off-state MDS-UPDRS3 scores. *Lt* left, *Rt* right

For susceptibility values of global analysis, the on-state MDS-UPDRS3 score correlated with left GP (r = 0.48, P = 0.036), right SN (r = 0.52, P = 0.023), and left SN (r = 0.52, P = 0.021) (Fig. 5).

**Fig. 6:**
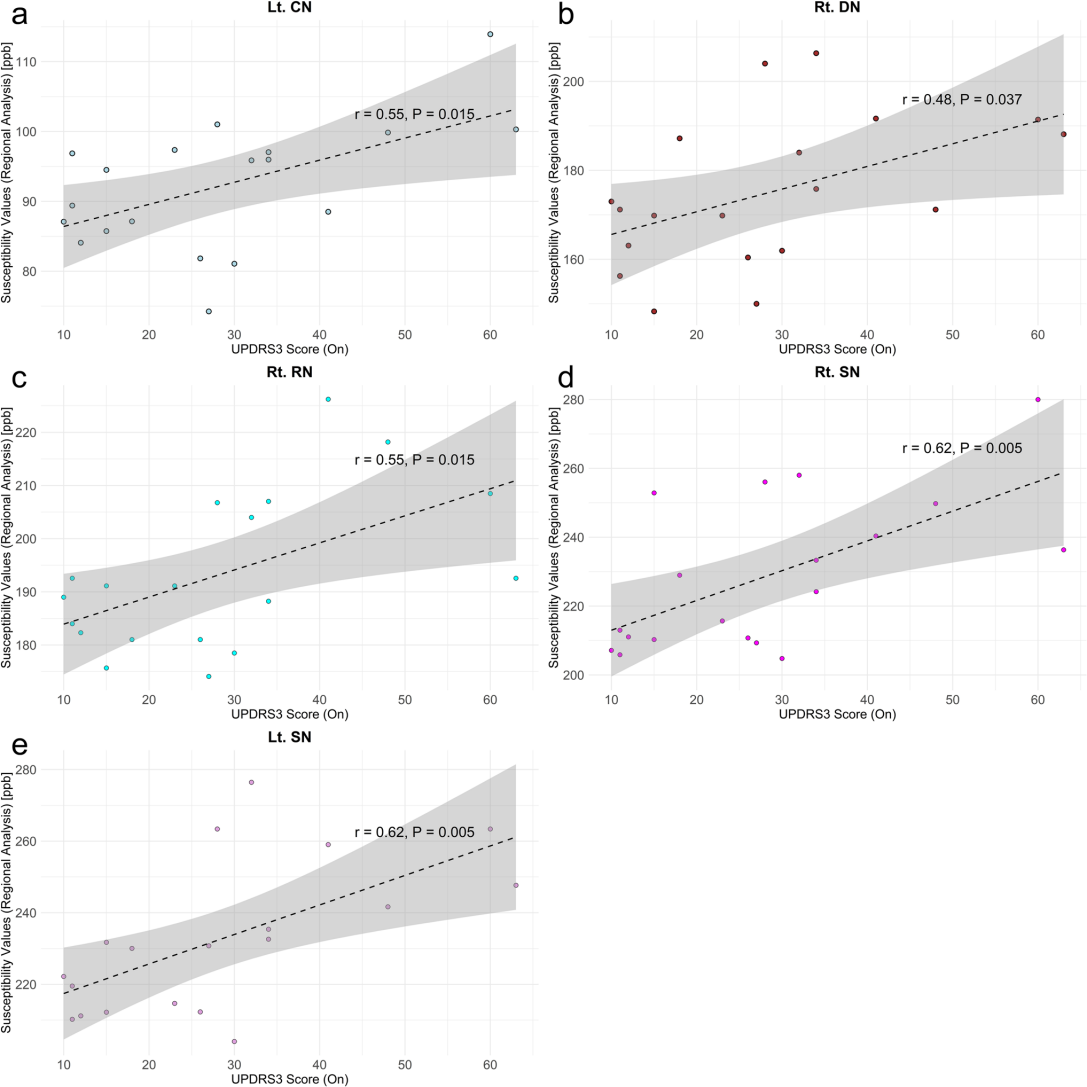
The relationship between regional susceptibility values and MDS-UPDRS3 scores showing the on-state MDS-UPDRS3 significantly correlated with **a** Lt. CN (r = 0.55, P = 0.015), **b** Rt. DN (r = 0.48, P = 0.037), **c** Rt. RN (r = 0.55, P = 0.015), **d** Rt. SN (r = 0.62, P = 0.005), and **e** Lt. SN (r = 0.62, P = 0.005). No susceptibility values were correlated with the off-state MDS-UPDRS3 scores. *Lt* left, *Rt* right

For susceptibility values in the regional analysis, the on-state MDS-UPDRS3 score showed significant correlations with left CN (r = 0.55, P = 0.015), right DN (r = 0.48, P = 0.037), right RN (r = 0.55, P = 0.015), right SN (r = 0.62, P = 0.005), and left SN (r = 0.62, P = 0.005) (Fig. 6).

In contrast, no susceptibility values were correlated with the off-state MDS-UPDRS3 scores.

## Discussion

The MTC and double flip angle multi-echo protocol has been well optimized to create a robust high quality imaging protocol for clinical use. To detect the loss of the N1 sign in PD patients, the characterization of the N1 shape in the SN, along with the comparison of iron content based on the presence or absence of the N1 sign, can serve as a biomarker to distinguish PD from HCs.

The swallow-tail sign may reflect the volume of N1, which is partly supported by our results of NM volume although the N1 is located within the SN pars compacta ventralis [35] and the intermediate and lateral portion of SN, and does not represent the entire SNpc. We assessed the N1 sign with the tSWI, or susceptibility mapping that avoids geometry-induced phase variation by using the source magnetic susceptibility as a mask [33]. Although NM volume may be most likely to represent the increased proton density associated with human dopaminergic neurons [21, 22], N1 sign and NM-MRI still has a potential to be used as clinically useful biomarker for PD, however, 3 out of 21 HCs showed no detectable N1 sign for unknown reasons, which warrants caution. NM volumes were not correlated with either of MDS-UPDRS3 scores in this study as previous reports [14, 36, 37]. Whether NM volumes on NM-MRI correlate with MDS-UPDRS3 scores remains controversial.

Susceptibility values of global analysis (CN, PT, and Pul) and regional analysis (PT, Pul, and TH) for HC were significantly higher than those of PD in this study. On the contrary, SN is the only structure showing elevated susceptibility values in both global and regional analyses with higher susceptibility values in PD patients when compared with the HC cohort in previous reports [10, 28]. Other DGMs in addition to SN showed elevated susceptibility values in PD [38]. However, the alterations in susceptibility were less consistent in the basal ganglia in PD: only 4 of 22 studies of PT showed a significant increase of susceptibility values in PD while one study reported significantly decreased susceptibility at PT in PD [39, 40].). Right TH in HC showed significantly higher susceptibility than that in non-demented PD. [41]. Although discrepancies exist, susceptibility values in the regional analysis showed significant correlations between the on-state MDS-UPDRS3 score and left CN, right DN, right RN, right SN, and left SN, suggesting that susceptibility values may reflect the severity of PD. Furthermore, iron deposition in other DGMs was instrumental in creating the optimal model for differentiating between PD and healthy controls in this study. The contribution of iron deposition should be further investigated in future studies.

There are several limitations in this study. First, the number of subjects is limited. This may partly be attributed to the absence of correlations between NM volume and MDS-UPDRS3 scores as well as between iron and SN in PD patients against HCs. Secondly, the number of patients in the early stages of PD —such as those with a disease duration of less than 5 years and/or Hoehn and Yahr stage 1 or 2— is relatively small. Third, patients with established diagnosis of PD were enrolled in this study. Early diagnosis of PD within the prodromal period is expected. Fourth, although the optimal model for distinguishing PD patients from HC achieved a high AUC of 0.99, the result may be affected by overfitting. Our findings should be interpreted with caution and considered primarily exploratory and hypothesis-generating. Further studies with larger cohorts are warranted to validate and expand upon these results. Fifth, the acquisition time of MTC and SWI is slightly long. NM-MRI and QSM can be performed using faster imaging sequences [15, 42]. Sixth, the reason of discrepancy of susceptibility values of DGM in both global and regional analyses between previous studies and this study remains unclear [10, 28]. In addition to differences of sample size, population (e.g., country of origin), other unidentified factors may also be involved. Seventh, susceptibility values from the side corresponding to the predominant symptoms were not analyzed in this study. No significant laterality differences were observed, possibly due to the relatively small number of patients with early stages of PD.

In conclusion, integrated analysis of PD-specific MR findings including N1 sign, NM volume, and iron content in the DGM was performed for each subject in this study. The best model consistently differentiated PD from HCs, achieving a high AUC.

## Supplementary Information

Below is the link to the electronic supplementary material.

Supplementary file1 (DOCX 773 KB)
