# Supplementary material for: Integrated evaluation of Nigrosome 1 sign, neuromelanin-sensitive MR and iron deposition
Source: Jpn J Radiol. 2025 Aug 18;44(1):24–34. doi: 10.1007/s11604-025-01858-7 (PMC12769993; doi:10.1007/s11604-025-01858-7)
Supplement: Supplementary file 1 — Supplementary file1 (DOCX 773 KB) [file 11604_2025_1858_MOESM1_ESM.docx]

**Supplementary Figure 1**

**
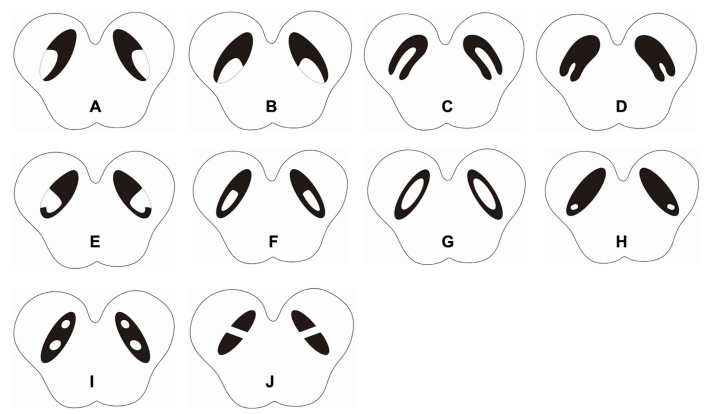
**

Cartoon illustration of the Nigrosome 1 variants. A-D in the ﬁrst row show the swallowtail sign variants due to the scanning plane or individual iron content in the normal Nigrosome 1. E-H in the second row show the loop or ovoid sign variants, and I-J in the third row show the Nigrosome 1 as a cluster or strip sign. Figure is used from the following article [10].

**Supplementary Figure 2**

**
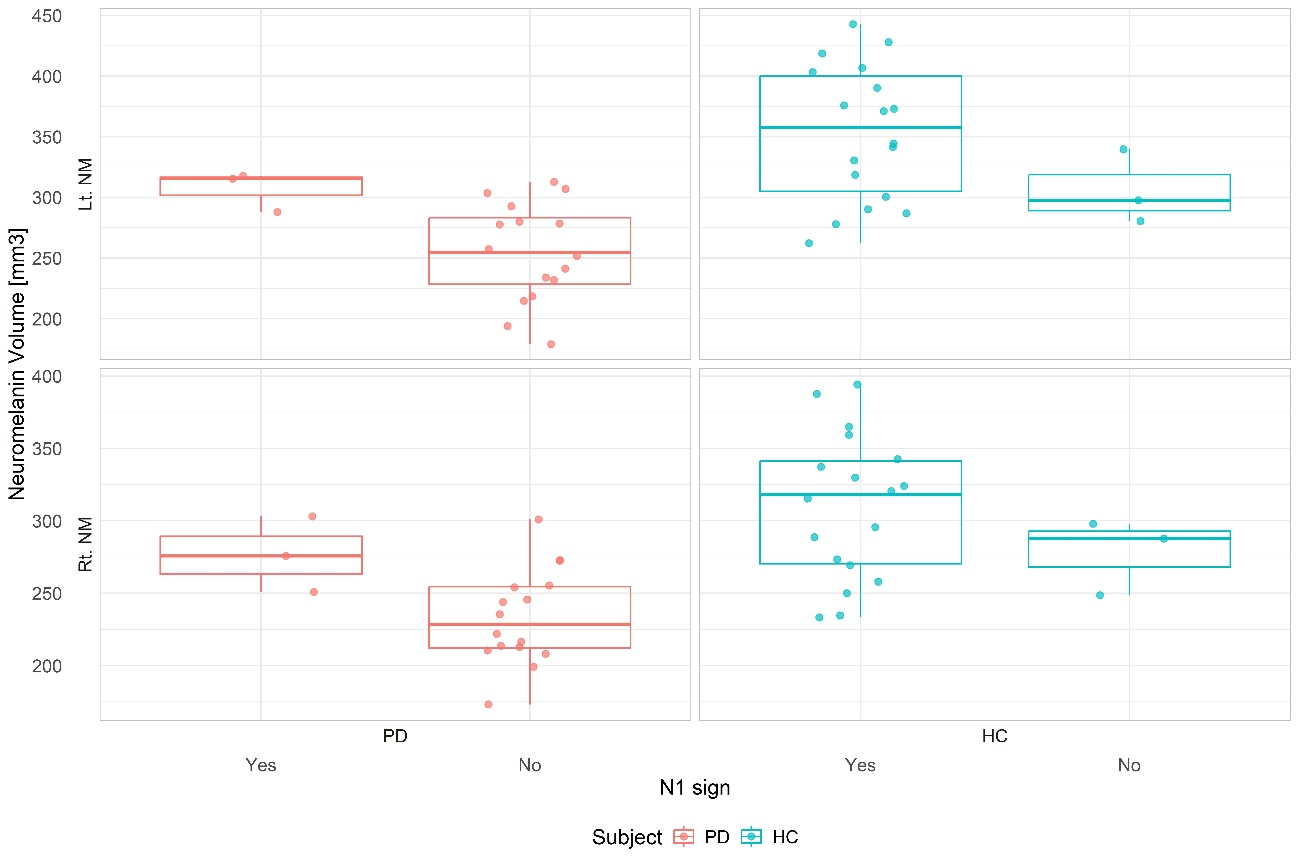
**

Box and whisker plots of neuromelanin volume are presented for individuals categorized by the presence or absence of the N1 sign (yes/no) and for patients with Parkinson's disease (PD) as well as healthy controls (HCs). Subjects with a positive N1 sign exhibit higher neuromelanin volumes.

**Supplementary Figure 3**

**
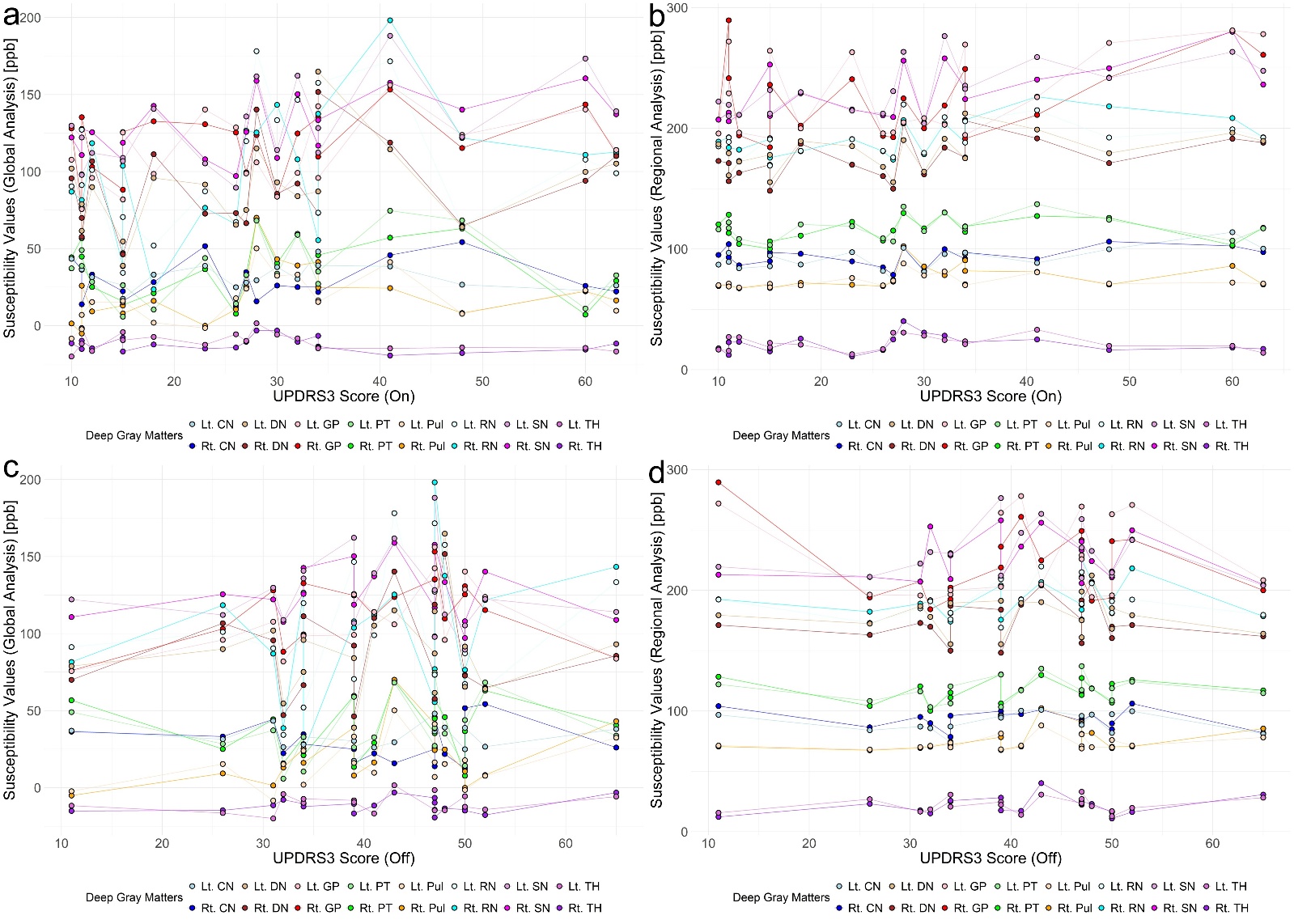
**

Susceptibility values of all VOIs from global analysis (a, c) and regional analysis (b, d) and MDS-UPDRS3 scores of the on-state (a, b) and the off-state (c, d) are shown. Susceptibility values that showed significant correlations with MDS-UPDRS scores are presented in Figure 5 and 6.

Note that the off-state score for one patient, whose on-state score was 60, was not recorded.

**Supplementary Annex**

**Methods**

Symptom laterality was assessed in patients with Parkinson’s disease (PD). When clear asymmetry was not observed, the average value of both sides was used for analysis.
Quantitative susceptibility values in the substantia nigra (SN) were compared between the symptom-dominant and non-dominant sides using both global and regional analyses.

**Results**

Among 19 patients with PD, 11 exhibited right-side dominance, 6 left-side dominance, and 2 showed no clear lateralization.

Global analysis (QSM):
SN susceptibility [ppb] — Dominant side: 125.6 [111.6, 142.0]; Non-dominant side: 122.5 [109.7, 139.8]; *P* = 0.85.

Regional analysis (QSM):
SN susceptibility [ppb] — Dominant side: 227.6 [211.4, 252.1]; Non-dominant side: 230.0 [211.4, 239.3]; *P* = 0.78.
